# Supplementary material for: iTRAQ-Based Quantitative Proteomics Analysis Reveals the Mechanism of Golden-Yellow Leaf Mutant in Hybrid Paper Mulberry
Source: Int J Mol Sci. 2021 Dec 23;23(1):127. doi: 10.3390/ijms23010127 (PMC8745438; doi:10.3390/ijms23010127)
Supplement: Supplementary file 1 [file ijms-23-00127-s001.zip › Supplementary Figures.pdf]

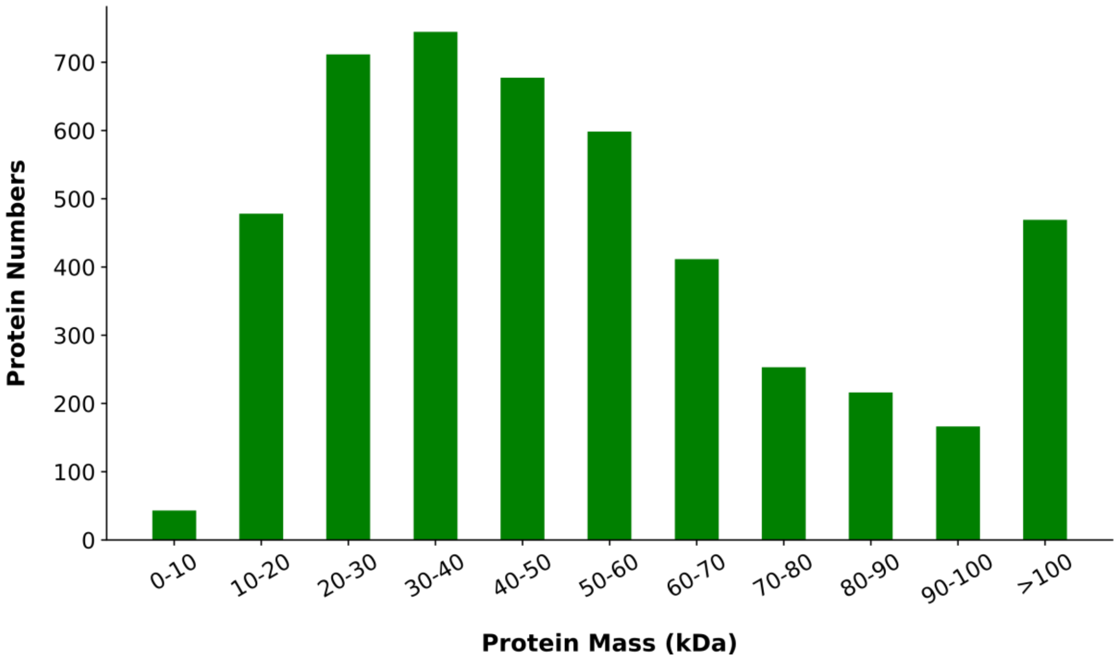

Figure S1. The distribution of protein mass in hybrid paper mulberry.

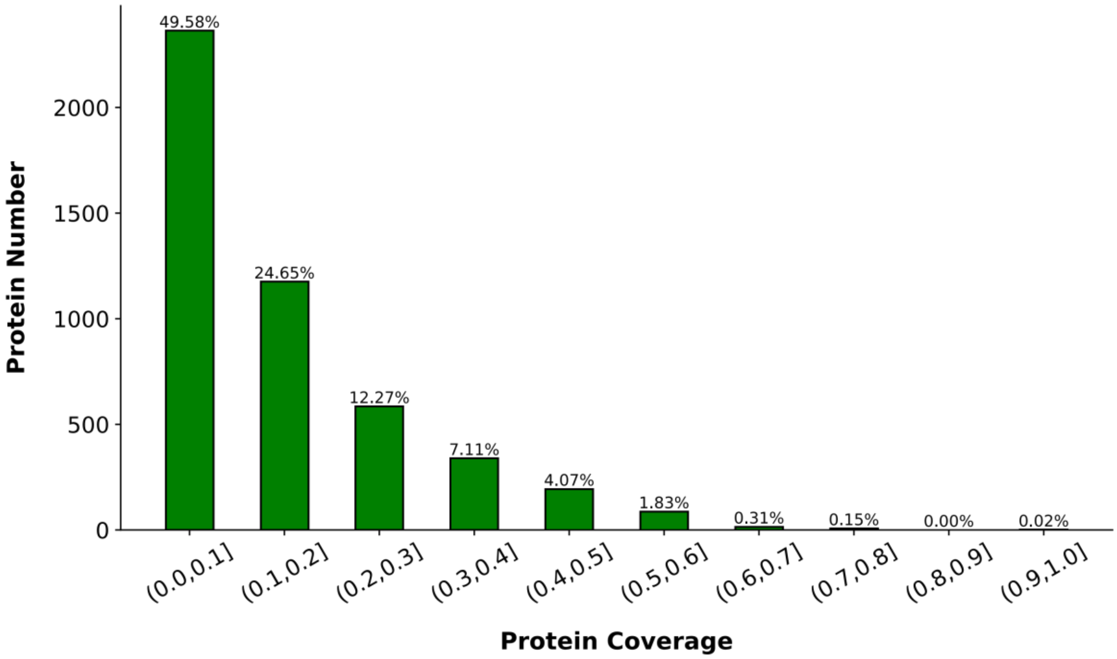

Figure S2. The distribution of peptide number in hybrid paper mulberry.

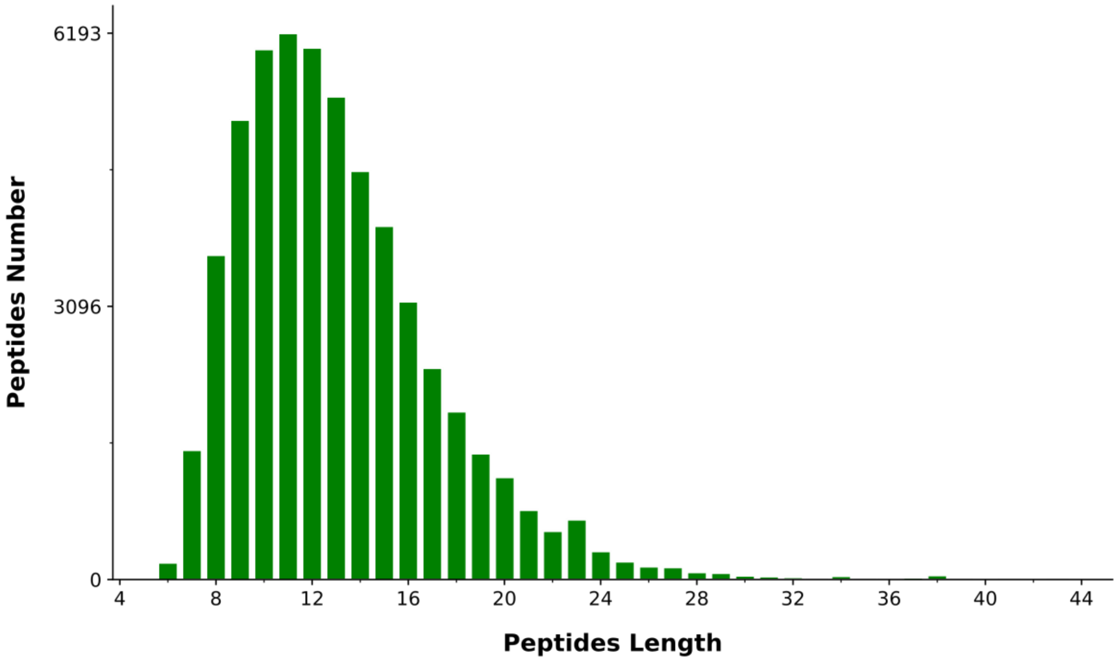

Figure S3. The distribution of peptide length in hybrid paper mulberry.

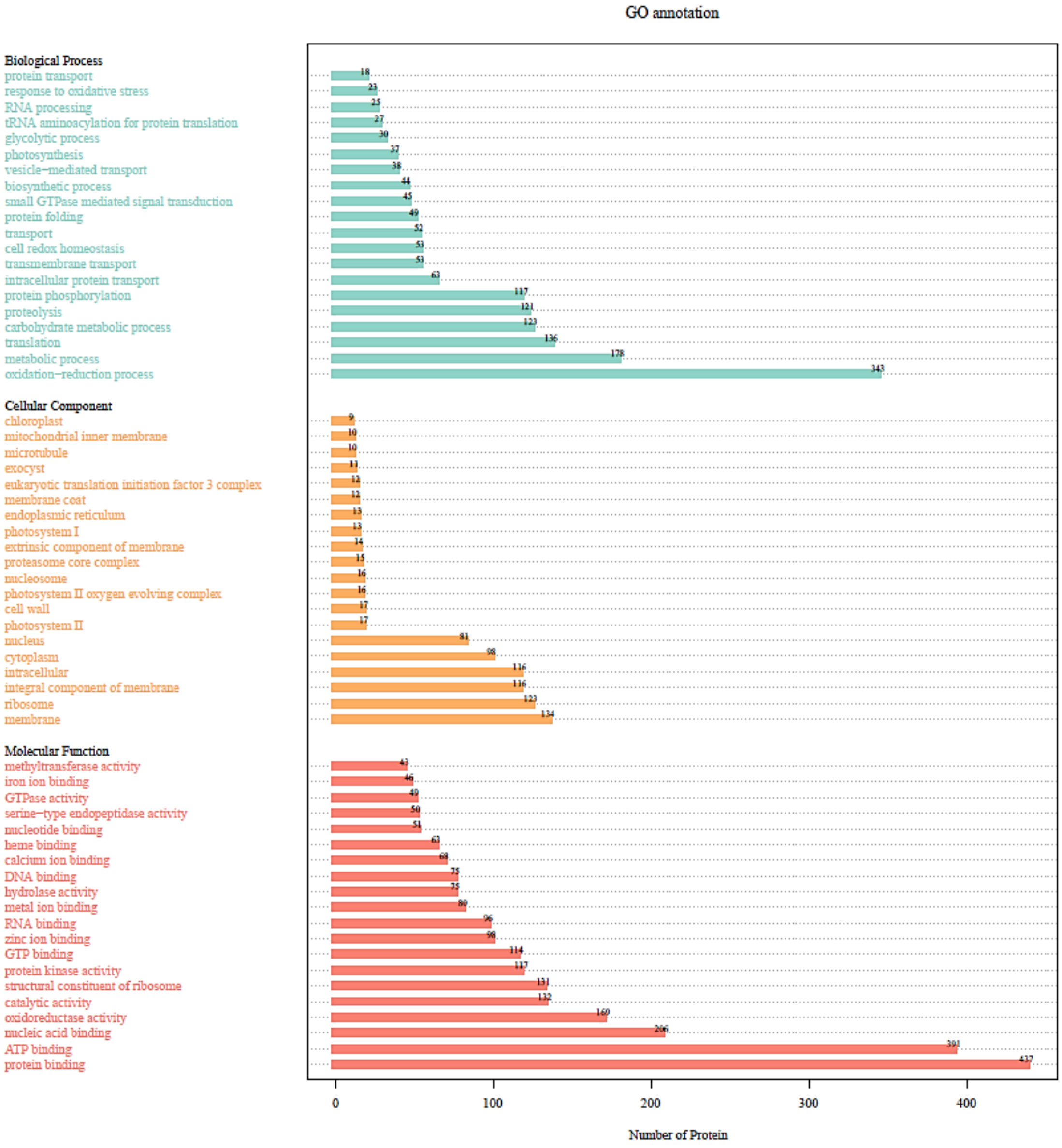

Figure S4. Gene ontology (GO) classification of proteins in hybrid paper mulberry.

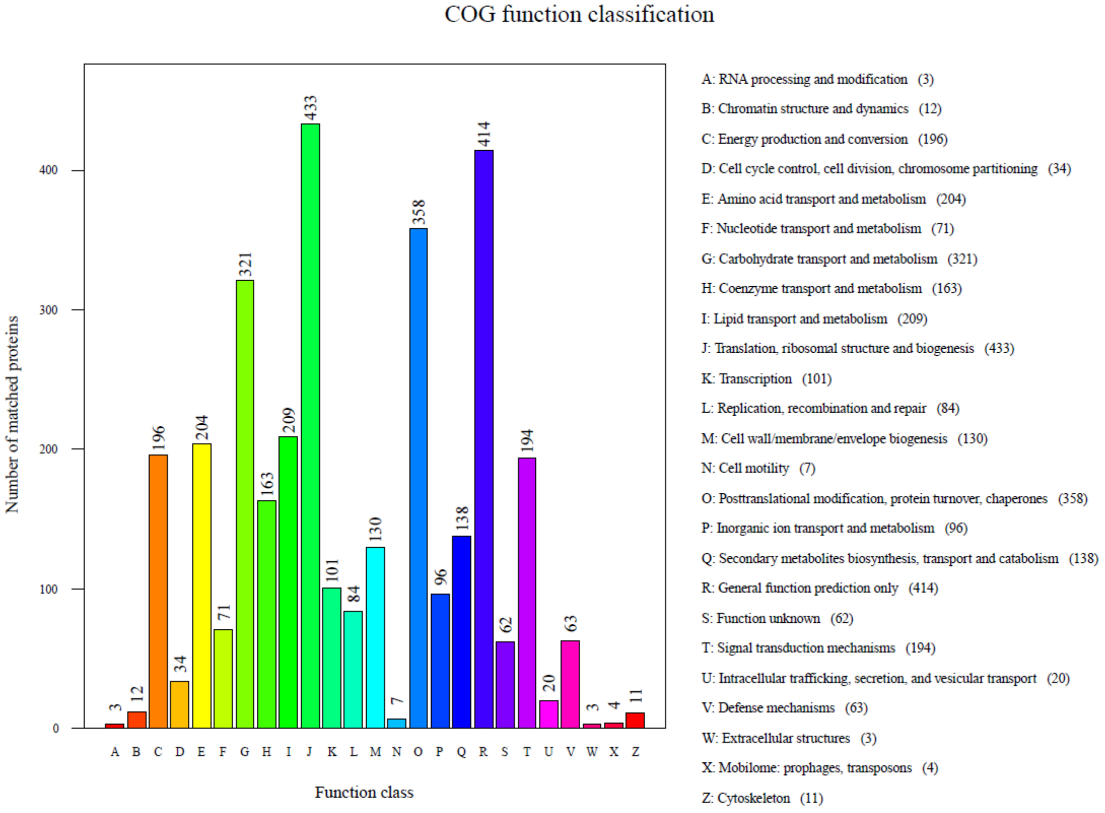

Figure S5. Clusters of Orthologous Groups of proteins (COG) classification of proteins in hybrid paper mulberry. A: RNA processing and modification; B: Chromatin structure and dynamics; C: Energy production and conversion; D: Cell cycle control, cell division, chromosome partitioning; E: Amino acid transport and metabolism; F: Nucleotide transport and metabolism; G: Carbohydrate transport and metabolism; H: Coenzyme transport and metabolism; I: Lipid transport and metabolism; J: Translation, ribosomal structure and biogenesis; K: Transcription; L: Replication, recombination and repair; M: Cell wall/membrane/envelope biogenesis; N: Cell motility; O: Posttranslational modification, protein turnover, chaperones; P: Inorganic ion transport and metabolism; Q: Secondary metabolites biosynthesis, transport and catabolism; R: General function prediction only; S: Function unknown; T: Signal transduction mechanisms; U: Intracellular trafficking, secretion, and vesicular transport; V: Defense mechanisms; W: Extracellular structures; X: Mobilome: prophages, transposons; Z: Cytoskeleton.

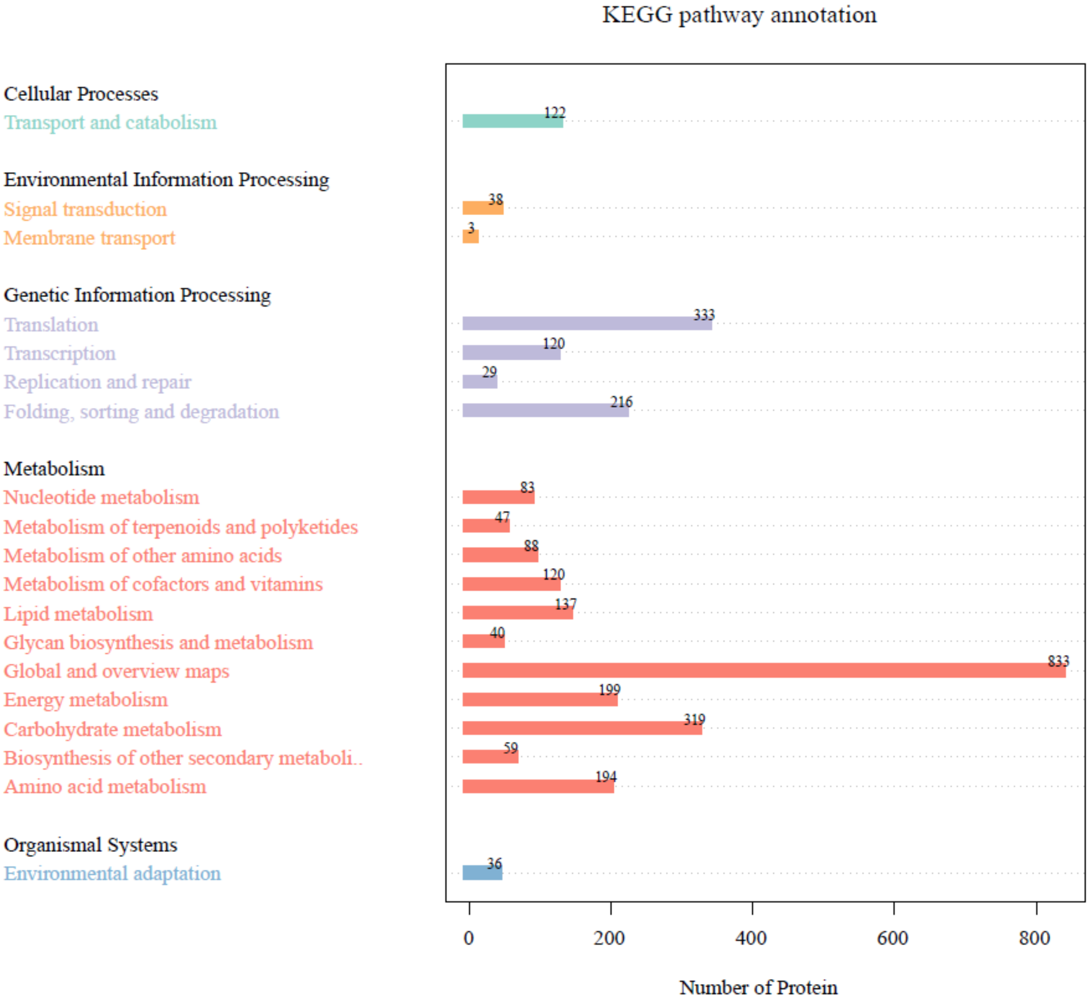

Figure S6. Pathways enrichment analysis of proteins in hybrid paper mulberry.
